# Local management strategies for inflammatory vaginitis in dermatologic conditions: Suppositories, dilators, and estrogen replacement

**DOI:** 10.1016/j.jdin.2022.09.004

**Published:** 2022-09-23

**Authors:** Katie Hinchee-Rodriguez, Amber Duong, Christina N. Kraus

**Affiliations:** a Department of Dermatology, University of California Irvine, Irvine, California; b School of Medicine, University of California Irvine, Irvine, California

**Keywords:** lichen planus, pemphigus, vaginal, Vulvar

## Therapeutic challenge

Prevalence of vaginal involvement in erosive lichen planus and pemphigus vulgaris has been reported in over 58% and up to 44% of patients, respectively.^1^^,^^2^ While most dermatologists are comfortable utilizing ultrapotent steroids on the vulva, less are comfortable with managing vaginal inflammation.

## Solution

**Fig 1:**
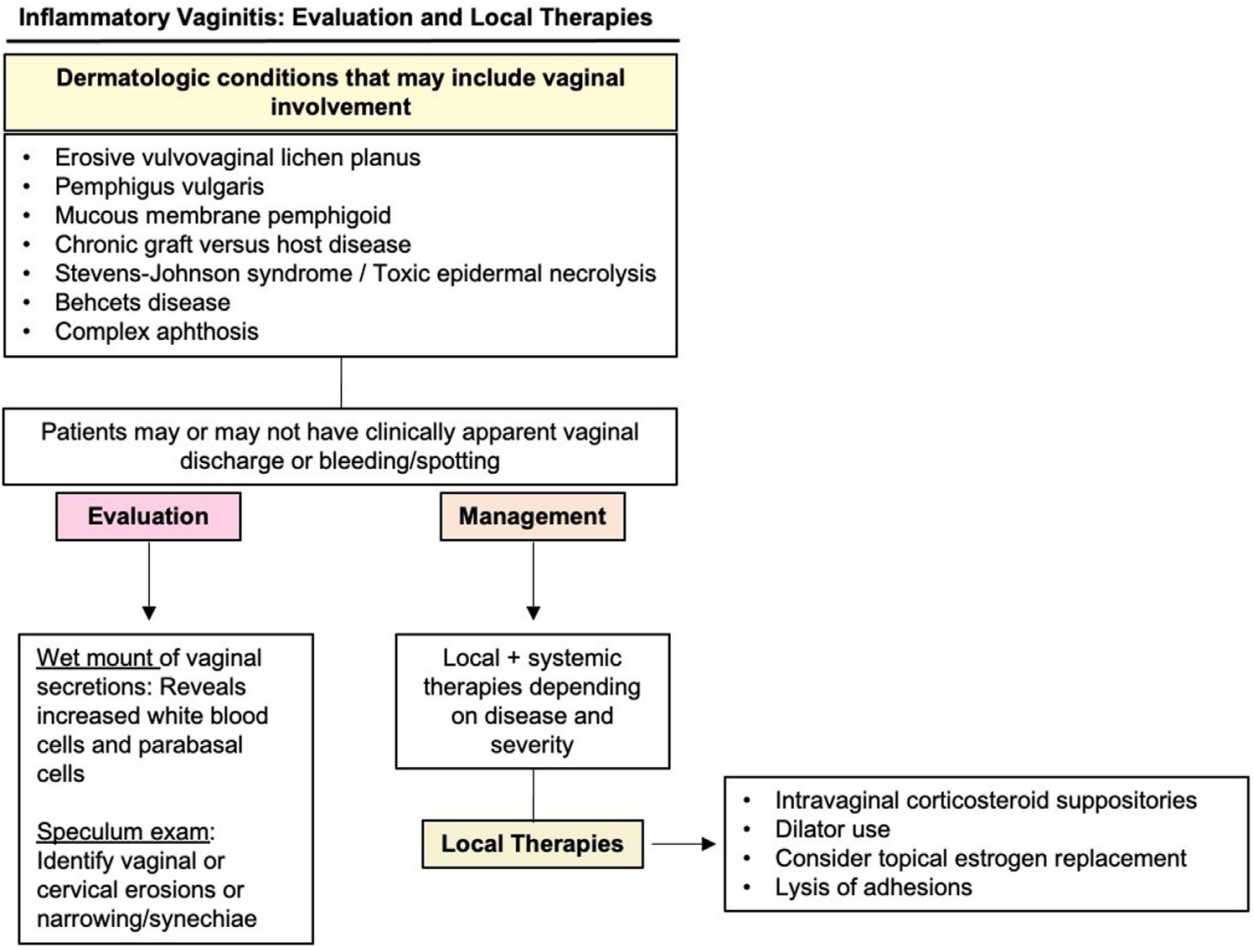
Inflammatory vaginitis: evaluation and management with local therapies.

Here, we highlight 3 local strategies to target inflammatory vaginal dermatoses and prevent scarring (Fig 1).

### Corticosteroid suppositories

We recommend starting with 25-mg hydrocortisone acetate suppositories, available as rectal suppositories. Patients should be counseled to use vaginally instead of rectally. Suppositories are initiated nightly for 2 to 4 weeks and decreased to every other night, or 2 to 3 times per week depending on disease control. Patients should be evaluated monthly, and suppositories should be tapered to the lowest dose/frequency that maintains disease remission.^2^ If more potent suppositories are required, hydrocortisone can be compounded at doses of 100 to 200 mg.

When prescribing suppositories, we recommend candidal prophylaxis, with topical antifungals 2 to 3 times per week or oral fluconazole 150 to 200 mg weekly.

### Vaginal dilators

Insertion of dilators 3 times, weekly, to prevent adhesions. Dilators can be ordered online and come in sets of varying sizes. Patients should use the largest dilator comfortable, and vaginal moisturizers and lubricants can be utilized.

### Estrogen replacement

Estrogen deficiency should be considered in postmenopausal patients as this can contribute to vaginal inflammation. Local estrogen therapy includes a ring, tablet, or cream. Generally, vaginal cream tends to have the least pain with insertion and is used nightly for 2 weeks with decrease to maintenance 1 to 3 times per week.

## Conflict of interest

None disclosed.
